# Assessment of All-Inside Sutures to the Posteromedial Capsule in Medial Meniscus Posterior Root Repair: Findings From a Retrospective Three-Dimensional Magnetic Resonance Imaging Study

**DOI:** 10.7759/cureus.73778

**Published:** 2024-11-15

**Authors:** Yuki Okazaki, Takayuki Furumatsu, Keisuke Kintaka, Yusuke Yokoyama, Masanori Tamura, Koki Kawada, Tsubasa Hasegawa, Toshifumi Ozaki

**Affiliations:** 1 Department of Orthopaedic Surgery, Okayama University Graduate School of Medicine, Dentistry and Pharmaceutical Sciences, Okayama, JPN; 2 Department of Orthopaedic Surgery, Japanese Red Cross Okayama Hospital, Okayama, JPN

**Keywords:** magnetic resonance imaging, medial meniscus extrusion, posterior root tear, pullout repair technique, three-dimensional reconstruction

## Abstract

Purpose: Medial meniscus (MM) posterior root tears (PRT) cause pathological medial extrusion (MMME) and posterior extrusion (MMPE), particularly during knee flexion, leading to rapidly progressive knee osteoarthritis. We investigated pre- and postoperative MM extrusion using three-dimensional open magnetic resonance imaging (MRI) following two pullout repair techniques: two simple stitches (TSS) and TSS with an additional all-inside suture to the posteromedial capsule (TSS-PM). We hypothesized that TSS-PM would decrease MM extrusion more effectively than TSS.

Methods: Thirty patients who underwent MM posterior root repair were retrospectively evaluated. TSS and TSS-PM techniques were used for pullout repair. Open MRI was performed at 10/90° of knee flexion preoperatively and three months postoperatively. MMME, MMPE, and MM extrusion volume (MMEV) were measured and compared between groups.

Results: At 90° of knee flexion, postoperative MMPE and MMEV were significantly decreased compared to preoperative values in both the TSS and TSS-PM groups. Furthermore, a significantly decreased ΔMMPE was observed in the TSS group compared to the TSS-PM group, whereas no significant difference was observed in ΔMMEV.

Conclusion: TSS and TSS-PM repair techniques helped decrease MMEV at 90° of knee flexion, whereas a significantly decreased ΔMMPE was observed in the TSS group at 90° of knee flexion. An additional all-inside suture at the posteromedial capsule may be insufficient to decrease MMEV and may negatively affect the decrease in ΔMMPE at 90° of knee flexion.

## Introduction

The meniscus stabilizes and protects the knee joint and absorbs shock [1]. Maintenance of hoop tension is one of the most essential functions of the meniscus, as it distributes the load stress and optimizes the contact pressure of the knee [2]. Recently, many studies have reported medial meniscus (MM) posterior root tears (PRT) and injuries within 1 cm of the MM posterior root attachment. According to a survey using open magnetic resonance imaging (MRI), MMPRT causes severe MM posterior extrusion (MMPE) during knee flexion compared with normal knees [3]. Additionally, MMPRT causes a loss of hoop tension and increases contact pressure on the knee joint, resulting in rapidly progressive osteoarthritis [4]. A biomechanical study reported that MMPRT caused a significant decrease in the contact area and increased pressure, similar to that in knees after total meniscectomy. In contrast, transtibial pullout repair restores these values within the normal range [5]. As MM medial extrusion (MMME) progresses shortly after MMPRT [6], early diagnosis and MM posterior root repair are recommended because of favorable clinical outcomes compared with conservative treatment or partial meniscectomy [2,7,8].

Several studies on MM extrusion have reported three-dimensional (3D) MM extrusion using open MRI. Open MRI can be used to evaluate the MMME, MMPE, and MM extrusion volume (MMEV) using 3D reconstructed images in the knee extended and flexed positions. Significantly decreased numbers of MMEV have been reported after MM posterior root repair, and postoperative clinical scores have been correlated with reduced posterior extrusion [7,9]. Further, several techniques have been reported to decrease the MM extrusion to achieve better clinical outcomes [7], such as centralization and additional all-inside sutures [10-12].

As discussed above, studies on MM extrusion using open MRI, additional techniques to decrease MM extrusion, and clinical outcomes after MM pullout repair have been widely conducted. However, no study has compared pre/post-MM extrusion (MMME, MMPE, and MMEV) at 10° and 90° of knee flexion following different techniques using open MRI. Several pullout repair techniques for MMPRT have been reported, including two simple stitches (TSS) and TSS with an additional all-inside suture to the posteromedial capsule (TSS-PM) [11,13]. Although favorable clinical outcomes have been reported for both techniques, few studies have compared them. We aimed to investigate the pre- and postoperative MM extrusion following two pullout repair techniques using 3D open MRI. We hypothesized that TSS-PM would be more effective than TSS in decreasing MM extrusion, as the additional suture allows for the grasped posteromedial portion of the MM to be pulled in more securely.

## Materials and methods

Patients

Data were collected retrospectively from medical records and MRIs. This study was approved by our institutional review board (Okayama University, approval No.: Rin 1857) and conducted in compliance with the Declaration of Helsinki. All patients provided written informed consent before participation.

This study included 40 patients who underwent pullout repair using MMPRT at our institution between April 2018 and June 2020. The TSS and TSS-PM techniques were used from April 2018 to October 2019 and from November 2018 to June 2020, respectively. All patients were preoperatively diagnosed with MMPRT based on MRI findings such as cleft, giraffe neck, and ghost signs [14,15]. The inclusion criteria were as follows: body mass index <30 kg/m^2^, femorotibial angle < 180°, osteoarthritis grades 0-2 (Kellgren-Lawrence classification), and Outerbridge grades I or II (mild cartilage lesions). Patients with other meniscal injuries, anterior cruciate ligament injuries, or previous ipsilateral knee surgeries were excluded. The TSS and TSS-PM groups included 13 and 17 patients, respectively, in the final cohorts.

Surgical procedures

A standard 4 mm diameter 30° arthroscope (Smith & Nephew, Andover, MA) with anterolateral and anteromedial portals was used. The MMPRT type was determined arthroscopically according to the meniscal root tear classification [16]. In every case, the percutaneous pie-crusting medial release was performed using a standard 18-gauge (1.2 × 40 mm) hypodermic needle (TERUMO, Tokyo, Japan) [17] to facilitate the procedure in the medial compartment.

TSS technique

**Figure 1:**
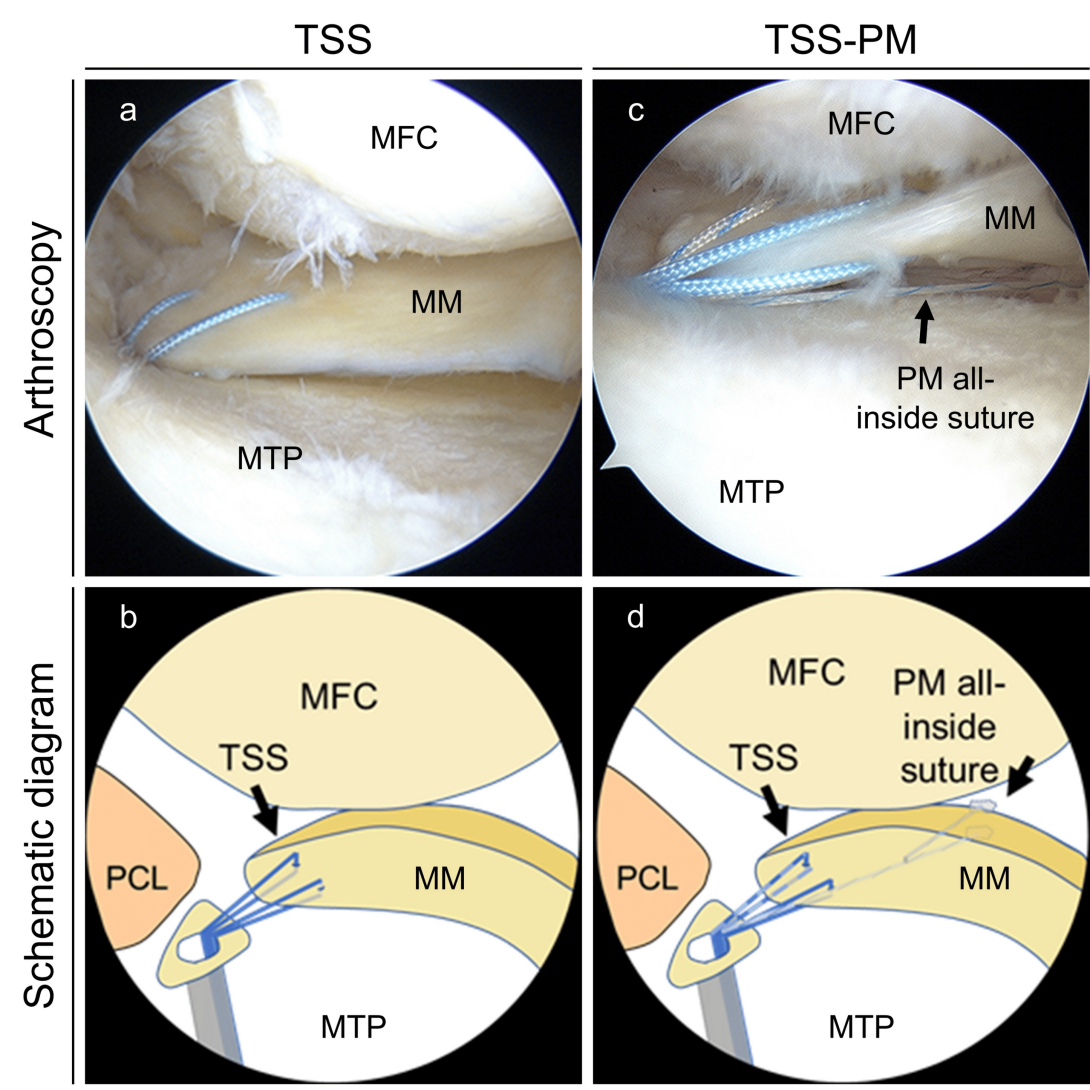
Arthroscopic findings and schematic diagram of the pullout repair (right knee) (a) The repaired medial meniscus posterior root tear (MMPRT) using the two simple stitches (TSS). (b) Schematic diagram of the TSS technique. (c) The repaired MMPRT using the TSS with posteromedial suture (TSS-PM). (d) Schematic diagram of the TSS-PM technique. MFC: Medial femoral condyle; MM: Medial meniscus; MTP: Medial tibial plateau; PCL: Posterior cruciate ligament; TSS: Two simple stitches; TSS-PM: Two simple stitches with posteromedial suture.

In the TSS group, two No. 2 UltraBraid (Smith & Nephew, Andover, MA) sutures were passed through the meniscal tissue using a Knee Scorpion suture passer (Arthrex, Naples, FL) as previously described [13]. The first suture was inserted into the inner area of the MM posterior horn, and the second was inserted into the outer area (Figure 1, Panels a and b).

TSS-PM technique

In the TSS-PM group, an additional all-inside suture, such as the FasT-Fix repair system (Smith & Nephew, Andover, MA), was used after TSS [11]. An additional all-inside suture was used to grasp a significant MM extrusion point in the posteromedial area of the MM [18]. Therefore, the first needle of the device was inserted into the inferior surface of the MM posteromedial segment, and the second needle was inserted directly into the articular capsule. The uncut-free end of the suture was pulled out from the bone tunnel, similar to the TSS, as previously described (Figure 1, Panels c and d) [11].

Fixation

After MM posterior root attachment was confirmed, a custom-made MMPRT guide (Smith & Nephew, Andover, MA) [19] was placed at the expected anatomic center of the attachment area (medial tibial plateau, posterior peak of the medial tibial eminence, and anterior border of the posterior cruciate ligament). A 2.4-mm guide pin was inserted at a 45° angle using the aiming device, and a 4.0-mm cannulated drill was used for over-drilling. After the removal of the inner guide pin and leaving the cannulated drill, two or three sutures were pulled out through the remaining drill using a suture retriever. After the expected tension (10, 20, or 30 N) was applied using a spring tensioner at 20° or 30° of knee flexion, tibial fixation was performed using a bioabsorbable interference screw and a cancellous screw (Meira, Aichi, Japan), as previously described [13]. In the TSS and TSS-PM groups, from April 2018 to October 2019, 30 N of tension was applied at 20° of knee flexion, whereas in the TSS-PM group from November 2019 to June 2020, 20 N of tension was applied at 30° of knee flexion.

Medial tibial slope angle measurement

Medial tibial slope (MTS) angle was performed on lateral radiographs by drawing two lines, as described by Brandon et al. [20], defined by the longitudinal axis of the tibia and the MTS, respectively. The MTS angle was defined as 90° minus the angle made by the intersection of the line of the longitudinal axis of the tibia and the MTS. The longitudinal axis of the tibia was defined by the line created by connecting the midpoint of the anteroposterior diameter of the tibia just inferior to the tibial tubercle and the midpoint of the anteroposterior diameter of the tibial shaft, measured no less than 5 cm distal to the first line.

3D MRI measurements

3D MRI examinations at 10/90° knee flexion were performed preoperatively and at three months postoperatively. Open MRI was performed using an Oasis 1.2 T system (Hitachi Medical, Chiba, Japan) with a coil at 10° and 90° of knee flexion under non-weight-bearing conditions, as previously described [21]. The standard sequences of the Oasis system included a sagittal proton density-weighted sequence (repetition time [TR]/echo time [TE], 1718/12) using a driven equilibrium pulse with a 90° flip angle and a coronal T2-weighted multi-echo sequence (repetition time/echo time, 4600/84) with a 90° flip angle. The slice thickness was 4 mm with a 0-mm gap. The field of view was 16 cm with an acquisition matrix size of 320 (phase) × 416 (frequency). As previously described, measurements were performed using an MRI-based meniscal sizing technique with sagittal views at 10° and 90° of knee flexion [11].

**Figure 2:**
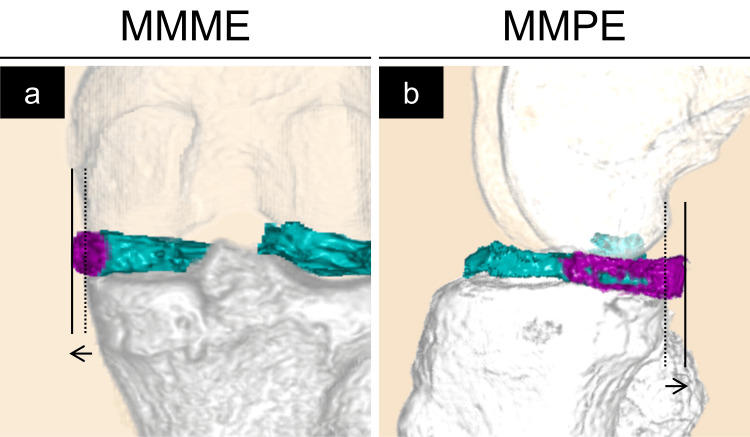
Three-dimensional reconstructed images of the medial meniscus (MM, green and purple), femur (transparent), and tibia (gray). The purple area represents the extruded MM beyond the tibial edge. (a) Posterior view: The dotted line shows the medial edge of the tibial plateau, and the solid line shows the medial edge of the MM. Medial extrusion of the MM was evaluated as the distance between them. (b) Medial view: The dotted line shows the medial edge of the tibial plateau, and the solid line shows the posterior edge of the MM. The posterior extrusion of the MM was evaluated as the distance between them.

**Figure 3:**
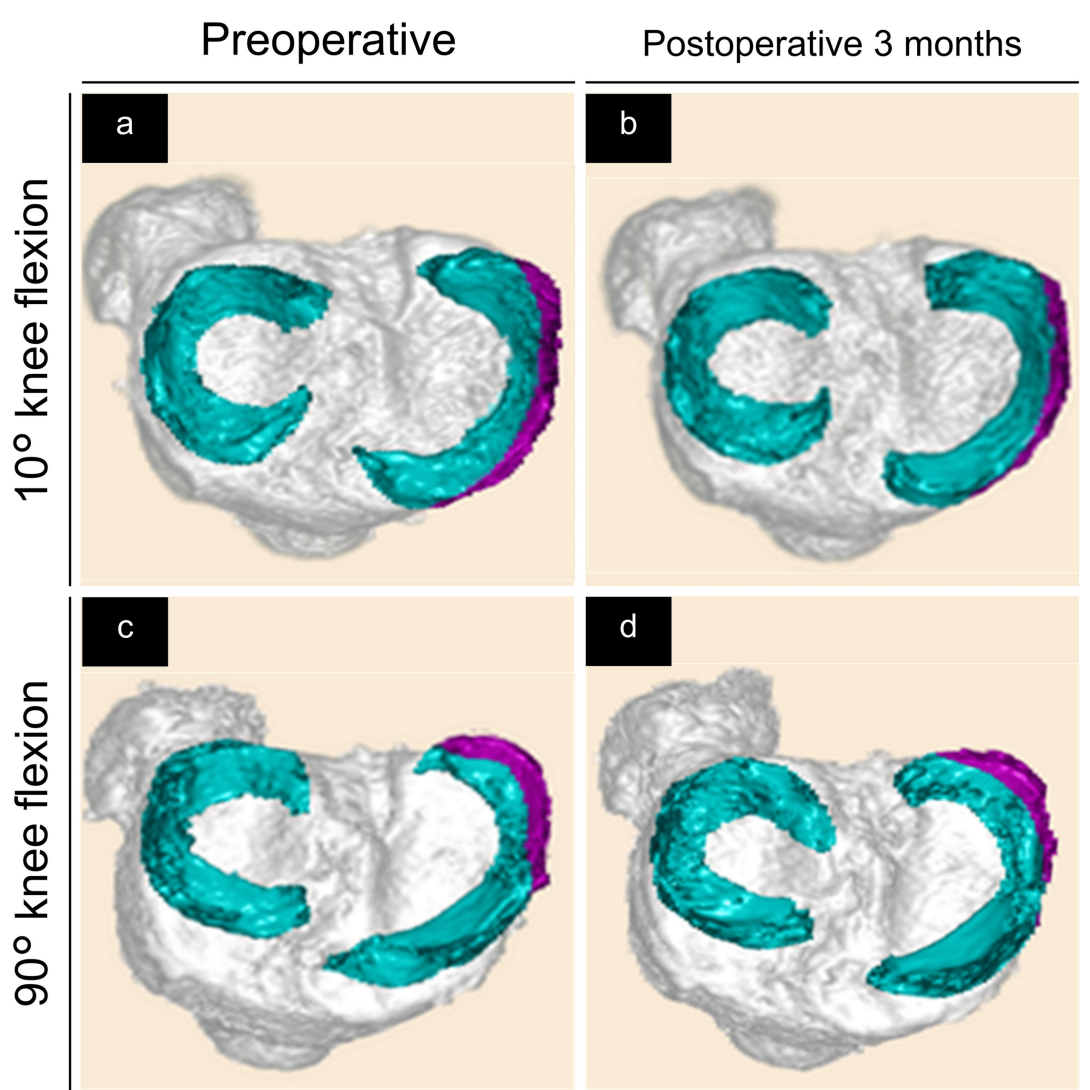
Preoperative and three months postoperative three-dimensional reconstructed images of the meniscus (green and purple) and tibia (gray). The purple area represents the extruded medial meniscus (MM) beyond the tibial edge—superior views in the two simple stitches group. (a) Preoperative image at 10° of knee flexion. (b) Three months postoperative image at 10°of knee flexion. (c) Preoperative image at 90° of knee flexion. (d) Three months postoperative image at 90° of knee flexion.

**Figure 4:**
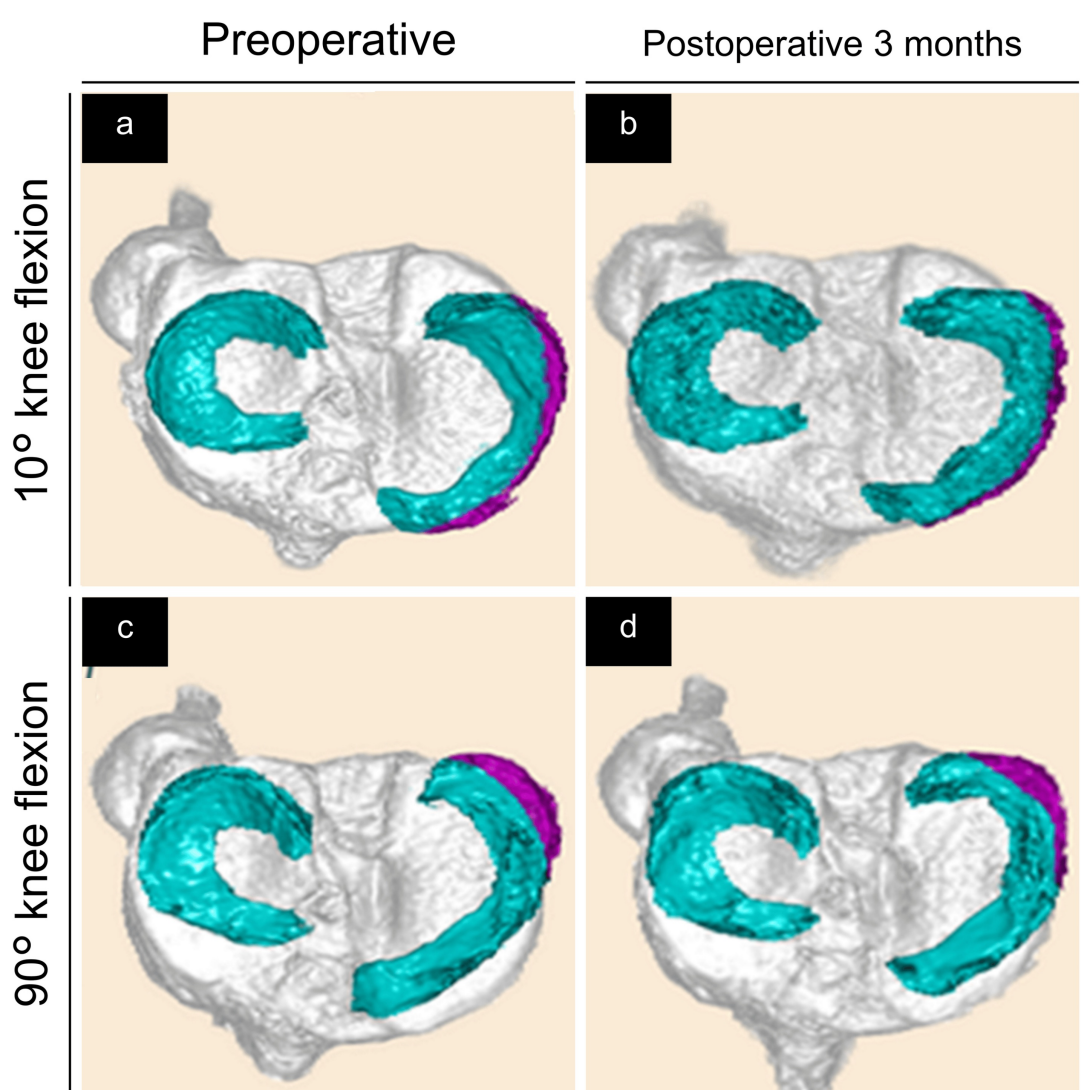
Preoperative and three months postoperative three-dimensional reconstructed images of the meniscus (green and purple) and tibia (gray). The purple area represents the extruded medial meniscus (MM) beyond the tibial edge—superior views in the two simple stitches with posteromedial suture group. (a) Preoperative image at 10° of knee flexion. (b) Three months postoperative image at 10° of knee flexion. (c) Preoperative image at 90° of knee flexion. (d) Three months postoperative image at 90° of knee flexion.

Figures 2-4 show the 3D-reconstructed images. The purple area indicates the extruded MM beyond the tibial edge. We evaluated the 3D MRI-based MMME, MMPE, MM volume (MMV), MMEV, and MMEV ratios as previously described [18]. The MMME and MMPE were defined as the distances from the medial and posterior edges of the tibia (excluding osteophytes), respectively, to the most extruded point of the MM (Figure 2). Using the posterior edge of the tibia as the standard, extrusions toward the posterior edge from the tibial edge were assigned positive values, whereas negative values indicated the absence of such extrusions (Figure 2, Panel b). MMV and MMEV were evaluated semi-automatically, followed by manual adjustment using the texture tracing technique [22,23] on a 3D image analysis workstation (SYNAPSE VINCENT®; Fuji Medical System, Tokyo, Japan). The MMEV ratio was calculated as MMEV divided by MMV to adjust for individual differences. In summary, the MMME, MMPE, MMV, MMEV, and MMEV ratios were measured and calculated at 10° and 90° of knee flexion both preoperatively and at three months postoperatively in both the TSS and TSS-PM groups (Figures 3, 4).

Statistical analysis

Data are presented as mean ± standard deviation. Statistical analyses and power calculations were performed using the EZR software (Saitama Medical Center, Jichi Medical University, Tochigi, Japan) [24]. Two orthopedic surgeons independently performed 3D MRI measurements in a blinded manner. Each observer performed each measurement twice, at intervals of at least two weeks. Intraobserver and interobserver reliabilities were calculated using the intraclass correlation coefficient (ICC). An ICC of ≥0.75 was considered excellent, 0.60-0.74 as good, 0.40-0.59 as fair, and <0.40 as poor. Intra- and intergroup differences were compared using the Wilcoxon signed-rank and Mann-Whitney U tests, respectively. Statistical significance was set at P < 0.05.

## Results

Table 1 shows the demographic and clinical characteristics of patients. No significant differences were observed between the two groups.

**Table 1:** Patient demographics and clinical characteristics Values are presented as mean ± standard deviation or number. * The Mann–Whitney U test was performed to compare intergroup differences. TSS: Two simple stitches; TSS-PM: Two simple stitches with posteromedial sutures.

Characteristics	TSS	TSS-PM	P-value
Number of patients	13	17	
Sex (men/women)	2/11	5/12	
Age (years)	65.2 ± 8.4 (60.1 - 70.3)	62.1 ± 8.6 (57.7 - 66.5)	0.65
Height (m)	1.56 ± 0.07 (1.52 - 1.60)	1.59 ± 0.08 (1.55 - 1.63)	0.47
Weight (kg)	60.7 ± 8.9 (55.3 - 66.1)	62.2 ± 9.0 (57.6 - 66.8)	0.67
Body mass index (kg/m^2^)	24.8 ± 2.7 (23.2 - 26.4)	24.6 ± 2.7 (23.2 - 26.0)	0.60
Femorotibial angle (°)	177.3 ± 1.7 (176.3 - 178.3)	177.1 ± 1.4 (176.4 - 177.8)	0.91
Medial tibial slope angle (°)	8.33 ± 2.31 (6.9 - 9.7)	8.80 ± 2.08 (7.7 - 9.9)	0.88
Root tear classification type 1/2/3/4/5	0/12/0/1/0	1/14/0/2/0	
Kellgren–Lawrence grade 0/1/2	0/6/7	0/8/9	

The results of the comparison between the preoperative and postoperative values, including MMME, MMPE, MMV, MMEV, and MMEV ratio, are presented in Tables 2, 3. At 10° of knee flexion, no significant differences were observed between the pre- and postoperative values in either the TSS or TSS-PM groups (Table 2). However, at 90° of knee flexion, the postoperative MMPE, MMEV, and MMEV ratios were significantly lower than the preoperative values in both the TSS and TSS-PM groups (Table 3).

**Table 2:** Comparison of three-dimensional magnetic resonance imaging-based data at 10° of knee flexion Values are presented as mean ± standard deviation. * The Wilcoxon signed-rank test was performed to compare intragroup differences. MMME: Medial meniscus medial extrusion; MMPE: Medial meniscus posterior extrusion; MMV: Medial meniscus volume; MMEV: Medial meniscus extrusion volume; TSS: Two simple stitches; TSS-PM: Two simple stitches with posteromedial sutures.

Characteristic	TSS			TSS-PM		
	Pre	Post	P-value	Pre	Post	P-value
MMME (mm)	3.4 ± 0.8 (2.9 – 3.9)	3.4 ± 1.4 (2.6 – 4.2)	0.84	3.7 ± 0.8 (3.3 – 4.1)	3.2 ± 0.9 (2.7 – 3.7)	0.09
MMPE (mm)	−2.4 ± 0.9 (-2.9 – -1.9)	–2.5 ± 0.8 (-3.0 – -2.0)	0.42	–3.6 ± 2.3 (-4.8 – -2.4)	–3.1 ± 2.4 (-4.3 – -1.9)	0.37
MMV (mm^3^)	2901 ± 599 (2539 – 3263)	2993 ± 573 (2647 – 3339)	0.19	3474 ± 413 (3261 – 3686)	3498 ± 606 (3186 - 3810)	0.96
MMEV (mm^3^)	866 ± 349 (654 – 1076)	923 ± 531 (602 – 1244)	0.74	1165 ± 412 (953 – 1376)	1065 ± 500 (808 – 1322)	0.14
MMEV ratio (%)	29.3 ± 9.3 (23.7 – 34.9)	29.8± 14.7 (20.9 – 38.7)	0.46	33.1 ± 10.4 (27.8 – 38.4)	30.3 ± 12.7 (23.8 – 36.8)	0.40

**Table 3:** Comparison of three-dimensional magnetic resonance imaging-based data at 90° of knee flexion Values are presented as mean ± standard deviation. * The Wilcoxon signed-rank test was performed to compare intragroup differences. MMME: Medial meniscus medial extrusion; MMPE: Medial meniscus posterior extrusion; MMV: Medial meniscus volume; MMEV: Medial meniscus extrusion volume; TSS: Two simple stitches; TSS-PM: Two simple stitches with posteromedial sutures.

Parameters	TSS			TSS-PM		
	Pre	Post	P-value	Pre	Post	P-value
MMME (mm)	2.2 ± 1.1 (1.5 – 2.9)	2.1 ± 1.1 (1.4 – 2.8)	1	2.3 ± 0.9 (1.8 – 2.8)	2.2 ± 1.7 (1.3 – 3.1)	0.16
MMPE (mm)	4.4 ± 1.3 (3.6 – 5.2)	2.2 ± 1.1 (1.5 – 2.9)	<0.01^*^	3.6 ± 1.2 (3.0 – 4.2)	2.3 ± 1.3 (1.6 – 3.0)	<0.01^*^
MMV (mm^3^)	3039 ± 541 (2712 – 3366)	3068 ± 490 (2772 – 3364)	0.27	3469 ± 502 (3210 – 3728)	3561 ± 588 (3259 – 3863)	0.35
MMEV (mm^3^)	1140 ± 363 (921 – 1359)	933 ± 439 (668 – 1198)	0.027^*^	1293 ± 422 (1076 – 1510)	889 ± 292 (739 – 1039)	<0.01^*^
MMEV ratio (%)	37.4 ± 8.8 (32.1 – 42.7)	30.0 ± 12.9 (22.2 – 37.8)	<0.01^*^	36.8 ± 9.6 (31.9 – 41.7)	25.4 ± 8.4 (21.1 – 29.7)	<0.01^*^

Tables 4, 5 present the results of comparison between the TSS and TSS-PM groups. No significant differences in Δ values were observed between the groups at 10° of knee flexion (Table 4). However, at 90° of knee flexion, a significantly decreased ΔMMPE was observed in the TSS group than in the TSS-PM groups (Table 5). TSS alone was better at decreasing the MMPE at 90° of knee flexion. Intra- and interobserver reliabilities for the MRI measurements at 10/90° were considered excellent, with an ICC of 0.89 (95% CI: 0.75-0.95) and 0.85 (95% CI: 0.67-0.95), respectively.

**Table 4:** Comparison of three-dimensional magnetic resonance imaging-based data at 10° of knee flexion Values are presented as mean ± standard deviation. The Mann–Whitney U test was performed to compare intergroup differences. MMME: Medial meniscus medial extrusion; MMPE: Medial meniscus posterior extrusion; MMEV: Medial meniscus extrusion volume; TSS: Two simple stitches; TSS-PM: Two simple stitches with posteromedial sutures.

Parameters	TSS	TSS-PM	P-value
ΔMMME (mm)	–0.062 ± 0.96 (-0.64 – 0.52)	–0.49 ± 0.75 (-0.88 – -0.10)	0.27
ΔMMPE (mm)	–0.14 ± 0.99 (-0.74 – 0.46)	0.45 ± 0.56 (0.16 – 0.74)	0.33
ΔMMEV (mm^3^)	0.057 ± 0.35 (-0.16 – 0.27)	–0.10 ± 0.23 (-0.22 – 0.02)	0.15
ΔMMEV ratio (%)	0.47 ± 12.80 (-7.3 – 8.2)	–2.80 ± 8.23 (-7.0 – 1.4)	0.43

**Table 5:** Comparison of three-dimensional magnetic resonance imaging-based data at 90° of knee flexion Values are presented as mean ± standard deviation. * The Mann–Whitney U test was performed to compare intergroup differences. MMME: Medial meniscus medial extrusion; MMPE: Medial meniscus posterior extrusion; MMV: Medial meniscus volume; MMEV: Medial meniscus extrusion volume; TSS: Two simple stitches; TSS-PM: Two simple stitches with posteromedial sutures.

Parameters	TSS	TSS-PM	P-value
ΔMMME (mm)	–0.054 ± 1.08 (-0.71 – 0.60)	–0.15 ± 0.68 (-0.5 – 0.2)	0.38
ΔMMPE (mm)	–2.20 ± 1.34 (-3.01 – -1.39)	–1.34 ± 1.85 (-2.30 – -0.39)	0.04*
ΔMMEV (mm^3^)	–0.21 ± 0.28 (-0.38 – -0.04)	–0.40 ± 0.35 (-0.58 – -0.22)	0.19
ΔMMEV ratio (%)	–7.3 ± 8.75 (-12.6 – -2.0)	–11.4 ± 10.6 (-16.9 – -6.0)	0.28

## Discussion

The most important finding of this study was that postoperative MMEV values were significantly decreased than preoperative values at 90° of knee flexion in both the TSS and TSS-PM groups, and ΔMMPE was significantly decreased in the TSS group compared with the TSS-PM groups. These results suggest that an additional all-inside suture to the posteromedial capsule has insufficient advantages and disadvantages in patients undergoing MMPRT.

MMPRT leads to MMME, resulting in the loss of hoop tension and increased contact pressure. Many studies have reported the relationship between postoperative MMME and clinical outcomes in patients undergoing MM posterior root repair. Some studies have linked MMME measurements to clinical outcomes and the progression of knee osteoarthritis [25,26]. However, other studies have reported that the MMME does not entirely correlate with clinical outcomes [27]. This paradox may have been caused by the definition of the MMME, which has been defined by many previous studies as the distance from the medial tibial edge to the medial edge of the MM in the coronal view on MRI at knee extension. However, the MMPRT can lead to severe posteromedial extrusion of the MM, particularly during knee flexion. Therefore, conventional MMME measurement methods cannot adequately evaluate posterior extrusion in knee flexion and may be insufficient for assessing postoperative MM extrusion, including MMEV, after pullout repair. In this study, conventional MMME, MMPE, and MMEV were evaluated. This suggests that this method can be used to assess the pathology of MMPRT more physiologically and accurately.

Previous studies have shown that the posterior MM in knees with MMPRT translates in a posteromedial direction during knee flexion [18]. To prevent posteromedial extrusion during knee flexion, we introduced an all-inside device into the posteromedial MM in the TSS-PM group [11]. Clinical outcomes and arthroscopic findings have already been reported, but this study is the first to evaluate 3D MM extrusion [28].

In both groups, the postoperative MMEV and MMPE at 90° of knee flexion significantly decreased compared with the preoperative values. A previous study demonstrated that the MMPE decreased at 90° knee flexion at three months postoperatively, which is consistent with our results [21,29]. Another study demonstrated that the MMEV was reduced at 90° knee flexion three months postoperatively, which is consistent with our results [7]. However, at 10° and 90° of knee flexion, there was no significant difference in ΔMMEV between the groups. Additionally, the ΔMMPE at 90° of knee flexion significantly improved in the TSS group compared to the TSS-PM group. Since MMPE is measured using a single sagittal slice, we could sensitively detect differences between groups. In contrast, MMEV may have been homogenized overall (as both groups showed improvement in MMME), which could explain why no significant difference was detected for the overall MMEV. This result suggests that an additional all-inside suture may have a disadvantage in reducing the postoperative MMPE. The force pulling MM toward the joint capsule by the additional all-inside suture was likely greater than the force pulling it inward during the pullout. As a result, MM was likely drawn closer to the joint capsule, which may have contributed to the increased MMPE observed in the TSS-PM group.

This study had several limitations. First, this was a retrospective study with a small sample size with a short-term follow-up as we wanted to evaluate the MM status before starting the daily activities and sports without limitation. Second, the relationship between MM extrusion and clinical outcomes, as well as meniscal degeneration, remains unclear, even though the short-term results in both groups were favorable [28,30]. Third, we could only evaluate the MM extrusion under non-weight-bearing conditions. Fourth, knee angle and tension during tibial fixation were not identical in all cases; however, it has been reported that a fixation angle of 20-30 degrees and a tension of 20-30 N consistently lead to favorable clinical outcomes [28]. Finally, biomechanical studies were not performed in patients who underwent either repair technique. Further studies on postoperative meniscal extrusion following the repair of MMPRTs, not only in 2D but also in 3D, with a more extended follow-up period are needed to improve our understanding of the pathology of MMPRTs and to assess the effectiveness of surgery with or without an additional technique to prevent MM extrusion.

## Conclusions

The TSS and TSS-PM repair techniques helped decrease MMEV at 90° of knee flexion, whereas a significantly decreased ΔMMPE was observed in the TSS group at 90° of knee flexion. An additional all-inside suture at the posteromedial capsule may be insufficient to decrease MMEV and may negatively affect the decrease in ΔMMPE at 90° of knee flexion. The TSS repair technique is simple and sufficiently effective in decreasing both MMEV and MMPE in acute MMPRT.
